# Adaptation of a routine immunoassay analyser for TSH measurement in dried blood spot and volumetric microsampling (Capitainer B50): evaluation for thyroid function testing in adults

**DOI:** 10.1016/j.plabm.2026.e00549

**Published:** 2026-07-04

**Authors:** Katarzyna Bornikowska, Anna Król, Magdalena Ostrowska, Konrad Kowalski, Wojciech Zgliczyński, Piotr Glinicki

**Affiliations:** a Department of Endocrinology, Centre of Postgraduate Medical Education, Warsaw, Poland; b EndoLab Laboratory, Centre of Postgraduate Medical Education, Warsaw, Poland; c Masdiag Laboratory, Masdiag Sp. z o.o., Warsaw, Poland; d Medical Department of Bioenergetics and Physiology of Exercise, Faculty of Health Sciences, Medical University of Gdansk, Dębniki 1, Gdańsk, 80-211, Poland

**Keywords:** TSH, Dried blood spot, DBS, Volumetric microsampling, Capitainer B50, Immunoassay, Thyroid function

## Abstract

**Background:**

Thyroid-stimulating hormone (TSH) is the primary biomarker for diagnosing and monitoring thyroid disorders. Although serum remains the reference matrix, microsampling approaches, including dried blood spots (DBS) and volumetric devices (CPT B50), may improve accessibility and compliance. This study evaluated the analytical and clinical performance of TSH measurement from DBS and CPT B50 compared with serum.

**Methods:**

Paired serum, DBS, and CPT B50 samples were collected from 246 adults undergoing thyroid evaluation. DBS and CPT B50 samples were eluted and analyzed using a chemiluminescence immunoassay (RUO, LIAISON® XL, DiaSorin). Analytical performance, recovery, repeatability, stability, and method agreement were assessed. Microsample results were converted to serum-equivalent concentrations using inverse Passing–Bablok regression equations. Performance was evaluated using Passing–Bablok regression and Bland–Altman analysis with predefined acceptance criteria (bias ≤ ±15%, CV ≤ 15%, recovery 85%–115%), guided by CLSI EP15-A3 and EP17-A2 guidelines.

**Results:**

Repeatability CVs ranged from 3.7% to 10.4%, and recovery was 97%–110%. DBS and CPT B50 showed strong agreement with serum, with mean bias of −0.10 mIU/L for CPT B50 and 0.24 mIU/L for DBS. Samples remained stable for up to 8 weeks at +4 °C and −20 °C. Thyroid status classification accuracy was 96% for DBS and 98% for CPT B50.

**Conclusions:**

TSH measurement from DBS and CPT B50 showed high analytical and clinical concordance with serum. Volumetric sampling improved reproducibility and stability, supporting its use in remote thyroid testing and therapy monitoring.

## 1 Introduction

Thyroid diseases are the most frequent endocrine disorders globally, with subclinical hypothyroidism affecting up to 15% of the adult population and overt hypothyroidism up to 0.8% [1,2]. Hyperthyroidism is less prevalent, with rates of approximately 0.8% in Europe and 1.2% in the United States [3]. Accurate diagnosis and effective monitoring rely on the measurement of thyroid-stimulating hormone (TSH) and free thyroxine (fT_4_) in serum.

TSH screening plays a crucial role in at-risk populations, including pregnant women, the elderly, and patients with autoimmune diseases, psychiatric conditions, or a history of neck irradiation [[4], [5], [6]]. Serum-based immunoassays—particularly third- and fourth-generation chemiluminescence immunoassays enable highly sensitive and specific TSH determination due to the use of dual monoclonal antibodies [7,8].

Despite significant advancements, serum TSH measurement is susceptible to interferences such as heterophilic antibodies (e.g., in pregnancy), macroTSH (subclinical hypothyroidism), and substances like biotin, as well as pharmacological agents (e.g., glucocorticoids, metformin, amiodarone) [[9], [10], [11], [12]]. Interpretation of TSH results also requires caution in specific populations, including children, the elderly, and individuals with non-thyroidal illness [13].

High-performance analytical techniques such as liquid chromatography–tandem mass spectrometry (LC–MS/MS) offer excellent specificity and the ability to quantify thyroid hormone isoforms. However, these methods are technically demanding and not routinely available in clinical laboratories [14]. In contrast, dried blood spot (DBS) and volumetric microsampling techniques (e.g., CPT B50) are sample collection approaches that enable minimally invasive, patient-centred testing, which can be combined with widely accessible immunoassay platforms.

DBS sampling offers practical advantages, including stability of analytes during transport, low sample volume, and suitability for home collection [15,16]. The introduction of volumetrically controlled microsampling devices, such as Capitainer (CPT B50), enables capillary blood collection in fixed volumes (10 or 50 μL depending on the device model). This approach prevents over- or under-sampling and enhances the analytical reproducibility of DBS testing. However, DBS analysis may be affected by preanalytical factors such as the haematocrit effect, incomplete drying, and matrix interferences, particularly at low analyte concentrations [17,18].

This study aimed to evaluate and validate the analytical and clinical performance of TSH measurement from dried blood spot samples—collected *via* standard cards and microsampling devices (Capitainer, CPT B50)— using chemiluminescence immunoassays on the LIAISON®XL (Diasorin) platform, compared to conventional serum testing.

## 2 Materials and methods

### 2.1 Study design and participants

The study included 246 adult participants aged 18–75 years who provided written informed consent. Inclusion criteria were absence of acute illness, recent blood transfusion, or active infection. Exclusion criteria comprised haematocrit <30% or >55% and known coagulopathies.

Of the 246 adults, 219 provided dried blood spot (DBS) samples and 83 provided volumetric microsamples using the Capitainer B50 (CPT B50) device. Some participants contributed both sample types. Samples were excluded if participants declined to provide additional material or if the DBS/CPT B50 samples were of insufficient quality for analysis (e.g., incomplete filling, uneven spreading, or contamination).

Each participant's serum, DBS, and/or CPT B50 results were paired for statistical analysis to assess agreement between matrices.

The study protocol complied with the Declaration of Helsinki.

### 2.2 Sample collection

Venous blood was collected into serum separator tubes, and capillary blood was obtained from finger-prick sampling performed by trained nurses. For conventional dried blood spot (DBS) collection, four drops (∼40 μL each) of capillary blood were applied to Ahlstrom TFN cards. For volumetric microsampling, the Capitainer B50 (CPT B50) device was used, which collects two separate 50 μL discs of whole blood. DBS samples were allowed to dry for at least 2 h at ambient temperature (20–25 °C) in horizontal position while CPT B50 samples were dried for at least 12 h before storage.

### 2.3 Analytical method

Serum and eluates from DBS and CPT B50 samples were analyzed using a chemiluminescence immunoassay (RUO, LIAISON® XL, DiaSorin, Italy).

The assay calibration was traceable to the WHO 3rd International Standard for TSH (IRP 81/565). DBS and CPT B50 spots were eluted in phosphate-buffered saline (PBS) containing 0.05% Tween-20 at 37 °C for 1 h with gentle shaking.

### 2.4 Precision and recovery

Precision and recovery were assessed using pooled serum and capillary samples covering the analytical range of TSH. For intra-assay (within-run) precision, five replicates of two samples with low and high TSH concentrations were analyzed within a single analytical run. For inter-assay (total) precision, the same samples were measured on five consecutive days (one run per day).

Recovery was evaluated by spiking low-TSH serum and DBS eluates with known concentrations of calibrator material and calculating the percentage of measured versus expected values.

Acceptance criteria were predefined as a coefficient of variation (CV) ≤15%, bias ≤15%, and recovery within the range of 85%–115%. The predefined bias acceptance limit was based on the desirable analytical performance specification for TSH derived from biological variation data available in the EFLM Biological Variation Database. These criteria were considered appropriate for the intended clinical application of TSH monitoring using microsampling approaches and were applied in accordance with CLSI EP15-A3 and EP17-A2 recommendations.

### 2.5 Pre-analytical stability

In order to assess the impact of pre-analytical factors on TSH concentrations in DBS samples, the study evaluated the effect of not drying DBS card after blood collection, as well as exposure to UV-C radiation. To evaluate the effect of incomplete drying, four drops (∼160 μL) of capillary blood were applied onto DBS cards and stored in sealed plastic bags at room temperature, 4 °C, or −20 °C for 12 h. Cards were subsequently dried for 3 h at ambient temperature and analyzed using the standard protocol. On the following day, the DBS cards were retrieved from the plastic bags and allowed to dry at ambient temperature for a period of 3 h. Thereafter, they underwent the standard protocol for the determination of TSH in DBS samples. The UV-C exposure test was conducted by positioning the dried DBS card at a distance of 20 cm from an 8 W UV-C germicidal lamp for a duration of 18 h.

### 2.6 DBS-related parameters

Validation of DBS-related parameters was conducted in accordance with the guidelines proposed by Capiau et al. (2019). All experiments were performed at a single physiological TSH concentration level. Test blood samples were prepared with defined haematocrit values by mixing serum (TSH 1.855 mIU/L) with red blood cells obtained from a healthy volunteer with blood group O Rh− to avoid serological incompatibility. The haematocrit of the resulting artificial blood was additionally cross-verified by centrifugation.

For the assessment of the haematocrit effect, samples were prepared at four Hct levels (0.3, 0.4, 0.5, and 0.6), covering the full physiological range, and applied to DBS cards at a fixed volume of 40 μL using an automatic pipette. To evaluate the effect of sample volume, blood was applied at volumes of 20, 40, 60, and 80 μL at a constant haematocrit of 0.4. To assess the effect of intra-spot distribution, blood was applied at a volume of 40 μL and a haematocrit of 0.4.

### 2.7 Method comparison and regression

Passing–Bablok regression analysis was performed using the original microsample and serum TSH concentrations to evaluate proportional and constant bias and to derive conversion equations.

Serum-equivalent concentrations were subsequently calculated using the regression equations:

TSHserum = (TSHCPT − 0.0025) / 0.0659

TSHserum = (TSHDBS − 0.0080) / 0.0199

Agreement between methods was further evaluated using Bland–Altman analysis, including calculation of mean bias, 95% confidence intervals (CI), and limits of agreement (LoA). Serum-equivalent concentrations were used for Bland–Altman analysis, reference interval transfer, and diagnostic classification.

### 2.8 Reference interval transfer and classification accuracy

The serum reference interval (RI) for TSH (0.3–3.6 mIU/L) was transferred to DBS and CPT B50 samples based on regression-derived correction factors. Diagnostic classification (euthyroid, hypothyroid, hyperthyroid) was compared between matrices using serum as the reference standard.

### 2.9 Sample stability

Stability of TSH in Capitainer B50 and DBS samples was assessed in secured zip-lock bags with desiccant (2 g silica gel) stored under different temperature conditions aimed to emulate real storage (4 °C and −20 °C) and transport (37 °C, 20 °C) conditions. Samples stored at 37 °C were additionally exposed to direct sunlight without glass barrier (UV-A radiation). Mean relative stability and CV values were calculated based on a representative sample measured in triplicate under each condition, with serum TSH reference of 1.32 mIU/L for CPT B50 and 2.37 mIU/L for DBS samples.

### 2.10 Statistical analysis

Statistical analysis was performed using Microsoft Excel and GraphPad Prism. Passing–Bablok regression was performed using XLSTAT (Addinsoft, Paris, France) integrated with Microsoft Excel. Agreement between serum and microsample TSH results was evaluated using Passing–Bablok regression and Bland–Altman analysis. Regression parameters (slope, intercept, and their 95% confidence intervals) were calculated. Sensitivity and specificity were calculated as TP/(TP + FN) and TN/(TN + FP), respectively. A deviation of ±15% was accepted as the predefined analytical equivalence limit.

## 3 Results

### 3.1 Analytical performance

Within-run and between-run precision studies performed on two control samples yielded coefficients of variation below 8% and 10%, respectively, meeting the predefined acceptance limit of CV ≤ 15% (Table 2). Recovery ranged from 91% to 108%, consistent across both DBS and CPT B50 matrices (Table 1).

**Table 1:** Thyroid-stimulating hormone (TSH) recovery quality control samples applied on Capitainer B50 devices. The CPT B50 values have been converted using the appropriate dilution factor.

Sample type	Sample	CRM		CPT B50 TSH (mIU/L)	Recovery (%)
		Target value	Min – Max		
CPT B50	QC 1	0.62	0.53 – 0.71	0.68	110%
	QC 2	7.33	6.23 – 8.43	7.09	97%
	QC 3	39.0	32.7 – 45.3	39.8	102%

**Table 2:** Imprecision of TSH measurement in DBS and CPT B50 samples.

Sample type	Sample	Mean conc. (mIU/L)	Intraday (within run)		Interday (total)	
			SD (mIU/L)	CV (%)	SD (mIU/L)	CV (%)
DBS	Patient 1	0.062	0.004	6.55%	0.005	7.02%
CPT B50	Pool-1	0.65	0.07	10.36%	0.07	11.38%
	Pool-2	7.03	0.30	4.24%	0.30	4.26%
	Pool-3	37.65	1.41	3.73%	1.32	3.49%

### 3.2 Method comparison with serum

**Fig. 1:**
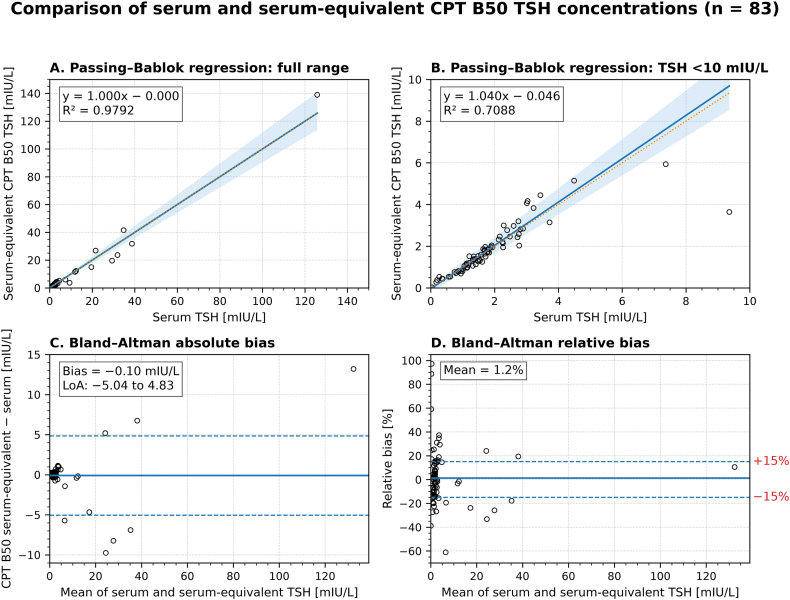
**Comparison of serum TSH concentrations (reference method) and CPT B50 serum-equivalent TSH concentrations (n = 83).** (A) Passing–Bablok regression across the full analytical range after conversion of CPT B50 results to serum-equivalent concentrations. (B) Passing–Bablok regression for serum TSH concentrations <10 mIU/L after conversion to serum-equivalent concentrations. (C) Bland–Altman plot showing absolute bias between serum and CPT B50 serum-equivalent concentrations. (D) Bland–Altman plot showing relative bias (%). The solid line represents the Passing–Bablok regression line; shaded bands indicate the 95% confidence interval of the regression line. Serum values are plotted on the x-axis. Dashed horizontal lines in panel D indicate predefined acceptance limits (±15%).

**Fig. 2:**
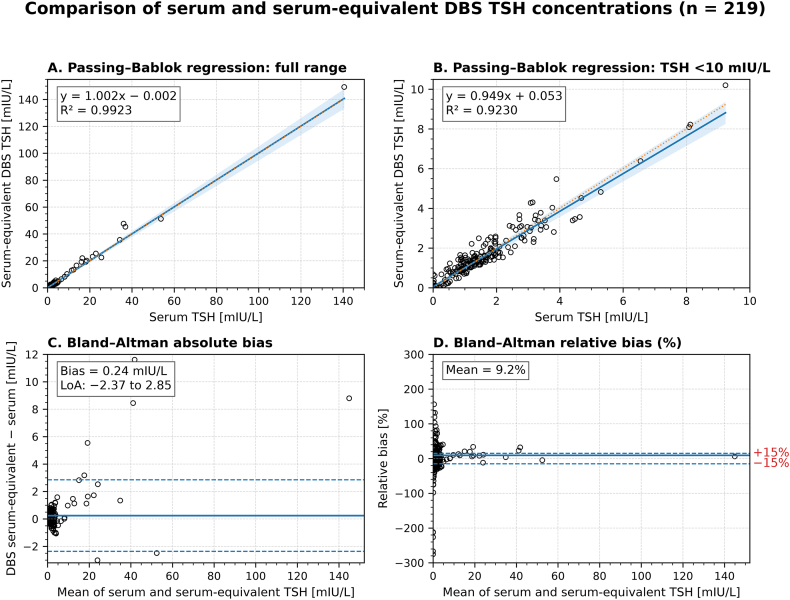
**Comparison of serum TSH concentrations (reference method) and DBS serum-equivalent TSH concentrations (n = 219).** (A) Passing–Bablok regression across the full analytical range after conversion of DBS results to serum-equivalent concentrations. (B) Passing–Bablok regression for serum TSH concentrations <10 mIU/L after conversion to serum-equivalent concentrations. (C) Bland–Altman plot showing absolute bias between serum and DBS serum-equivalent concentrations. (D) Bland–Altman plot showing relative bias (%). The solid line represents the Passing–Bablok regression line; shaded bands indicate the 95% confidence interval of the regression line. Serum values are plotted on the x-axis. Dashed horizontal lines in panel D indicate predefined acceptance limits (±15%).

Passing–Bablok regression analysis demonstrated excellent agreement between serum and serum-equivalent microsample TSH concentrations, as illustrated in Fig. 1A–D and 2A–D.

Mean relative bias was +1.2% for CPT B50 and + 9.2% for DBS, remaining within the predefined acceptance criterion of ±15%.

Absolute Bland–Altman analysis showed a mean bias of −0.10 mIU/L with limits of agreement ranging from −5.04 to 4.83 mIU/L for CPT B50 and 0.24 mIU/L with limits of agreement ranging from −2.37 to 2.85 mIU/L for DBS.

### 3.3 Pre-analytical factors

**Fig. 3:**
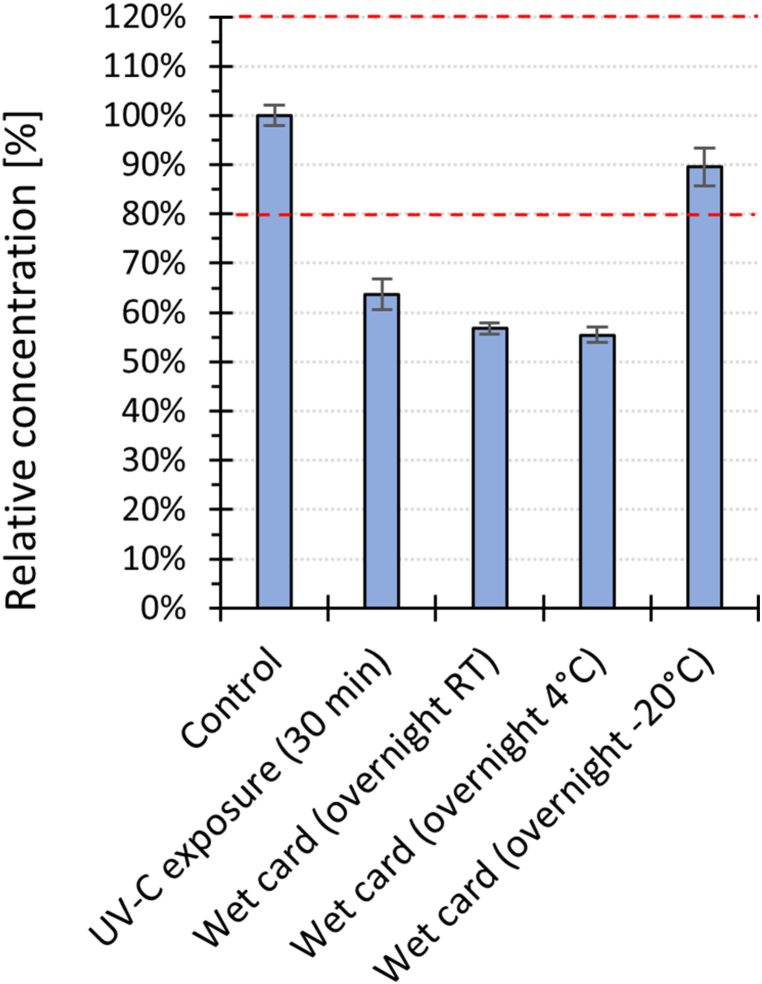
Effect of pre-analytical factors on TSH concentrations in DBS samples, including UV-C exposure and storage of undried blood spots. Results are expressed as percentage change relative to baseline values.

The results of the impact of pre-analytical factors on TSH measurement from DBS samples are summarised in Fig. 3. Research findings have demonstrated the importance of rapid and effective drying of the DBS card subsequent to the extraction of capillary blood for the purpose of maintaining TSH stability. It has been demonstrated that leaving the card undried overnight at ambient temperature or 4 °C results in a substantial decrease (−45%) in the TSH concentration within the sample. The decrease in TSH concentration observed in samples stored under these conditions may be attributable to the activity of proteolytic enzymes and/or a redistribution of blood within the card, where the cellular components of blood begin to degrade. This degradation manifested as a visible halo effect on the DBS cards, becoming immediately apparent upon removal from the foil pouches. However, once the cards had dried, this effect became less pronounced. This emphasises the necessity for meticulous sample qualification for TSH testing and for minimising the DBS card exposure to elevated humidity.

The results of the study demonstrate that deliberate (for the purpose of card disinfection) or accidental exposure to UV-C should be avoided, as it leads to probable TSH degradation even when the card exposed to UV-C radiation is properly dried.

### 3.4 Sample stability

**Fig. 4:**
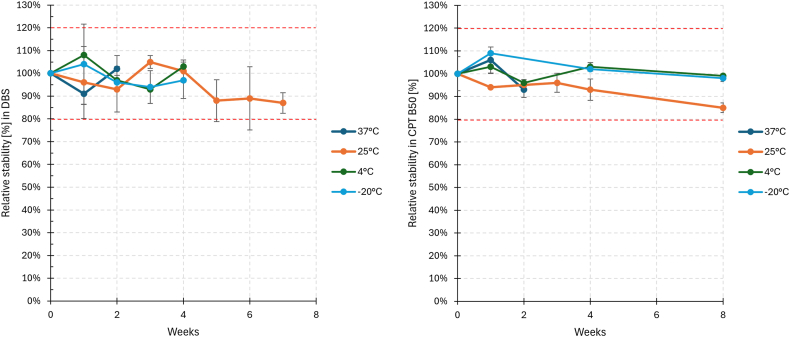
Stability of TSH in DBS (A) and Capitainer B50 (B) samples. Acceptance criterion: within ±20% of concentration at day 0. Dashed lines indicate predefined acceptance limits (±20%). Results are expressed as percentage of baseline (day 0).

The stability of TSH in Capitainer B50 and DBS samples was assessed under different temperature conditions for up to 8 weeks (Fig. 4). Both matrices showed excellent stability when stored at +4 °C and −20 °C, maintaining over 95% of baseline TSH concentration throughout the study period. Samples kept at 25 °C remained within acceptable limits for the first 4 weeks but showed a gradual decline thereafter. In contrast, samples exposed to 37 °C and direct sunlight demonstrated a marked decrease in recovery, with TSH concentrations falling below 90% after 2 weeks. These results confirm that appropriate drying and storage at cool or frozen temperatures are essential to preserve analyte integrity and ensure reliable TSH measurement in remote testing settings. Intermediate time points were not included due to limited sample availability.

### 3.5 DBS-related parameters

**Fig. 5:**
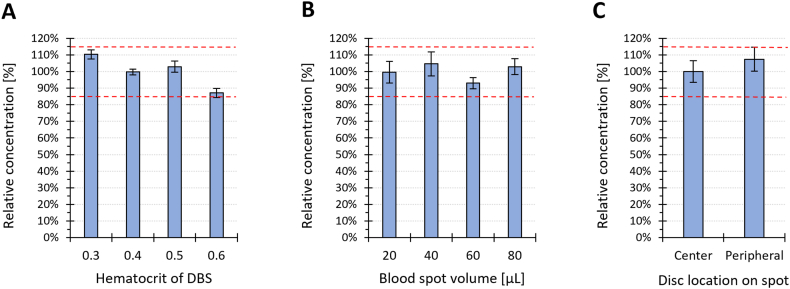
Evaluation of DBS-related parameters affecting TSH measurement according to Capiau et al. (2019). (A) Effect of haematocrit on TSH concentration at a constant sample volume (40 μL). (B) Effect of blood spot volume on TSH concentration at a constant haematocrit (Hct 0.4). (C) Effect of intra-spot distribution (volcano effect) on TSH concentration. Results are expressed as percentage deviation from reference values. Dashed lines indicate predefined acceptance limits (±15%).

Studies examining the influence of DBS-dependent parameters indicated no significant correlation between the size (volume) of the blood spot and its distribution, and the concentration of TSH in the DBS. At the same time, a slight inverse correlation was observed between the concentration of TSH and the haematocrit (Fig. 5). This is an expected result, given that TSH is found exclusively in serum, the relative amount of which decreases as haematocrit increases. Despite this apparent trend, it should be emphasised that the results obtained for the haematocrit effect meet the acceptance criteria adopted by Capiau et al. (2019).

No consistent upward or downward trend was observed across tested parameters, and all results remained within predefined acceptance limits.

### 3.6 Reference interval transfer

The serum reference interval (RI) of 0.3–3.6 mIU/L was transferred to DBS and CPT B50 using regression-based recalculation. Derived serum-equivalent RIs were 0.40–3.47 mIU/L for DBS and 0.26–3.53 mIU/L for CPT B50. These values were comparable with published DBS TSH RIs reported by Morris et al. (2022) [19].

### 3.7 Classification accuracy

Detailed diagnostic performance parameters are presented in Table 3. For DBS, sensitivity and specificity were 85%–93% and 95%–99%, respectively, across hypo- and hyperthyroid categories. These findings confirm high concordance with serum-based classification and support the clinical applicability of DBS-based sampling.

**Table 3:** Summary clinical validation parameters for TSH assays in DBS samples. Calculated based on DBS and serum TSH concentrations obtained both from healthy individuals or patients with compensated thyroid function during treatment with L-thyroxine (n = 185) and patients without thyroid function compensation (n = 61).

Parameter	Hyperthyroidism (n = 30)	Hypothyroidism (n = 31)	Hyper- or hypothyroidism (n = 61)
Sensitivity	85.2%	93.3%	89.5%
Specificity	98.9%	96.8%	95.1%

## 4 Discussion

Our study provides comprehensive validation of TSH measurement using dried blood spots (DBS) and volumetric microsampling (CPT B50) on the LIAISON XL platform (DiaSorin, Italy). Both approaches demonstrated analytical and clinical performance consistent with serum-based measurements, meeting predefined acceptance criteria. These results align with and extend previous reports by Schakelaar et al. (2023), who confirmed the feasibility of DBS-based TSH testing using an automated high-throughput immunoassay analyser (Atellica IM, Siemens, Germany) [20]. Similarly, Morris et al. (2022) established DBS-derived reference intervals for TSH in both adults and neonates, further supporting the clinical utility and stability of DBS-based thyroid testing [19]. Early studies on DBS-based TSH measurement in neonatal screening programs have demonstrated long-standing analytical reliability and clinical applicability, supporting the robustness of DBS methodology in endocrine diagnostics [[21], [22], [23]].

Together, these studies corroborate the robustness of DBS sampling for thyroid function assessment and demonstrate its potential to expand access to hormone testing beyond conventional laboratory settings.

In this study, evaluation of both conventional DBS cards and the Capitainer B50 volumetric device enabled a broader assessment of microsampling technologies. Volumetric sampling offered improved reproducibility by controlling applied blood volume and reducing haematocrit-related variability—limitations inherent to traditional DBS. This advantage was confirmed by the lower imprecision and bias observed for CPT B50 compared with DBS (CVs ≤10% vs ≤ 15%, respectively), supporting the superior reproducibility of volumetric sampling. These data demonstrate that controlled sample volume improves analytical precision, which is particularly important for decentralized testing. However, results obtained for CPT B50, both in terms of analytical performance and other validation parameters, outperformed those of both routinely used methods and previously published data.

Pre-analytical factors proved critical for assay performance. The method showed consistent results across physiological haematocrit values (35%–50%). The so-called ‘volcano effect’ increased apparent TSH concentration at the periphery of blood spots by up to approximately 22%, which is consistent with previous observations of analyte distribution heterogeneity within DBS matrices. Importantly, such deviation is unlikely to affect clinical classification owing to the broad decision range of TSH. Stability testing demonstrated preservation of TSH concentrations for up to eight weeks under refrigerated or frozen conditions, whereas elevated temperature or incomplete drying resulted in substantial analyte loss (>40%). Placement of undried DBS cards in sealed plastic bags caused a >45% signal decrease, only partially mitigated by desiccant use. UV-C exposure led to an average 36% reduction in measured TSH, highlighting the importance of adequate drying and handling before transport.

Method-specific reference intervals were established to facilitate clinical implementation. Using Passing–Bablok regression, the serum reference interval of 0.3–3.6 mIU/L was transferred to DBS (0.40–3.47 mIU/L) and volumetric samples (0.26–3.53 mIU/L), confirming clinical equivalence after regression-based adjustment (Table S1, Fig. S1). These data confirm that both DBS and volumetric microsampling can accurately reproduce serum TSH values within clinically acceptable limits, supporting their application in remote thyroid monitoring and patient self-sampling.

Overall, the analytical validation met the predefined acceptance criteria established a priori (bias ≤15%, CV ≤ 15%, recovery 85%–115%), guided by CLSI EP15 and EP17 guidelines. Relative bias showed increased variability at low TSH concentrations, which is expected due to the mathematical instability of percentage differences near zero values. Therefore, interpretation focused primarily on absolute bias and clinically relevant concentration ranges. From a clinical perspective, the method achieved classification accuracy exceeding 95% for both DBS and CPT B50 samples, supporting the reliability of both microsampling approaches for routine TSH monitoring.

The predefined bias acceptance criterion was selected according to the desirable analytical performance specification for TSH derived from the EFLM Biological Variation Database, ensuring that the validation targets reflected clinically relevant analytical quality requirements.

Clinical validation confirmed high diagnostic agreement between DBS- and CPT-derived TSH results and conventional serum testing (Table 3). Sensitivity and specificity exceeded 80% and 95%, respectively (Table 3), supporting the clinical applicability of both microsampling approaches for thyroid function monitoring. Stability studies confirmed that DBS and volumetric samples remain stable for extended periods under refrigerated or frozen conditions, ensuring analytical integrity during transport and storage.

In summary, both conventional DBS and volumetric microsampling (CPT B50) offer analytically robust and clinically reliable alternatives to serum-based TSH testing, paving the way for decentralized and patient-driven thyroid monitoring.

## 5 Limitations

Our study has several limitations. All participants had haematocrit values within sex-specific reference ranges; therefore, the effect of extreme Hct (e.g., anemia or polycythemia) could not be assessed. Volumetric samples were collected only from capillary blood, while serum was venous, so paired comparisons of matrix origin were not performed. Finally, only a 50 μL volumetric device was tested; smaller volumes (20–30 μL) were not evaluated. Future studies should address performance across different haematocrit extremes, sample origins, and metered volumes.

Overall, our findings confirm that both DBS and volumetric microsampling (CPT B50) are reliable tools for TSH measurement, with analytical and clinical performance comparable to serum. These minimally invasive approaches are well suited for diagnostic testing, routine monitoring, and treatment optimization in thyroid disease, and they support future applications in remote and home-based sampling strategies.

An additional limitation relates to the analytical variability accepted for method validation. Although the predefined acceptance criteria (bias ≤15%, CV ≤ 15%, recovery 85–115%) were considered appropriate for routine TSH monitoring, such variability may influence patient classification when TSH concentrations are close to clinically relevant diagnostic or therapeutic decision limits. Therefore, results obtained near established cut-off values should be interpreted with caution and, where clinically indicated, confirmed using conventional venous serum testing to minimize the risk of misclassification.

## 6 Conclusions

TSH measurement from DBS and volumetric microsampling (CPT B50) devices on the LIAISON® XL platform is a reliable alternative to serum testing, providing strong agreement, robust analytical performance, and excellent clinical performanceexcee. These minimally invasive approaches are suitable for routine monitoring of thyroid disease, including thyroxine dose adjustment, and offer practical benefits for remote or resource-limited settings.

## CRediT authorship contribution statement

**Katarzyna Bornikowska:** Data curation, Investigation, Writing – original draft. **Anna Król:** Methodology. **Magdalena Ostrowska:** Formal analysis. **Konrad Kowalski:** Methodology. **Wojciech Zgliczyński:** Supervision. **Piotr Glinicki:** Supervision.

## Declaration of competing interest

The authors declare that they have no known competing financial interests or personal relationships that could have appeared to influence the work reported in this paper.

## Data Availability

Data will be made available on request.
