# Supplementary material for: Adaptation of a routine immunoassay analyser for TSH measurement in dried blood spot and volumetric microsampling (Capitainer B50): evaluation for thyroid function testing in adults
Source: Pract Lab Med. 2026 Jul 4;51:e00549. doi: 10.1016/j.plabm.2026.e00549 (PMC13351441; doi:10.1016/j.plabm.2026.e00549)

**Table S1. Method-specific reference intervals for TSH.**
The local laboratory serum reference interval (0.3–3.6 mIU/L) was transferred to DBS and volumetric samples using Passing–Bablok regression. Transformed lower and upper limits are shown alongside the reference method.

| **Method** | **Lower limit** | **Upper limit** | **Approach** |
| --- | --- | --- | --- |
|  | **(mIU/L)** | **(mIU/L)** |  |
| Serum | 0.30 | 3.6 | Local laboratory reference interval |
| DBS | 0.40 | 3.47 | Passing–Bablok transfer (this study) |
| Volumetric | 0.26 | 3.53 | Passing–Bablok transfer (this study) |

**Figure S1. Comparison of serum, DBS, and volumetric reference intervals for TSH.**
Horizontal bars indicate the lower and upper limits of the reference interval for serum (black), DBS (red), and volumetric dried blood samples (blue). Intervals for DBS and volumetric samples were obtained by regression-based transfer of the serum reference range.


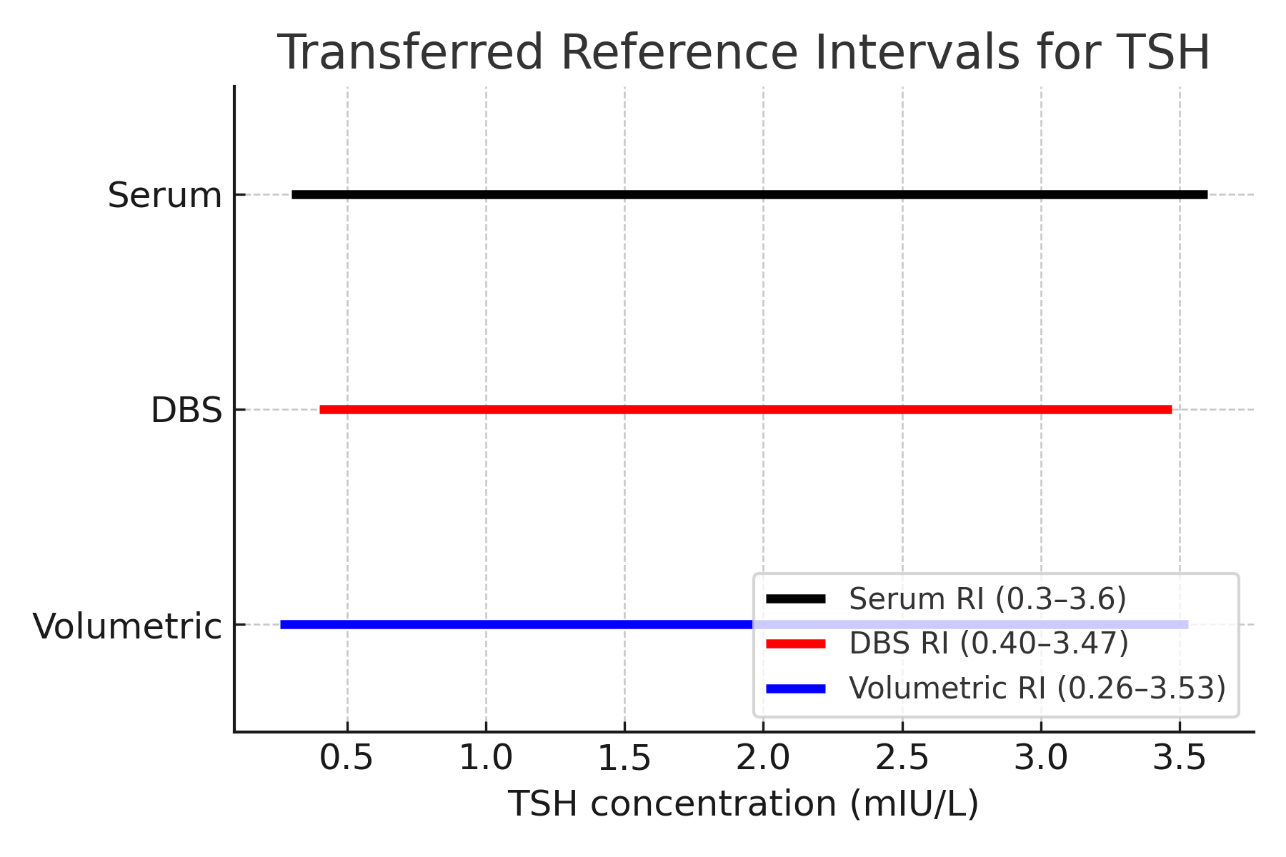


**Figure S2. Workflow of TSH measurement from dried blood spots (DBS).**Capillary blood was collected by finger prick and applied onto DBS collection cards. After drying for at least 2 hours at room temperature, cards were stored at 4–8 °C in sealed bags with desiccant. Circular punches (Ø 4.6 mm) were excised and subjected to extraction in phosphate-buffered saline (PBS) containing 0.05% Tween-20 for 60 minutes at 37 °C with shaking. Extracts were subsequently analyzed for TSH using a chemiluminescence immunoassay (RUO, LIAISON® XL, DiaSorin). Extracted samples were subsequently used for calculation of serum-equivalent TSH concentrations
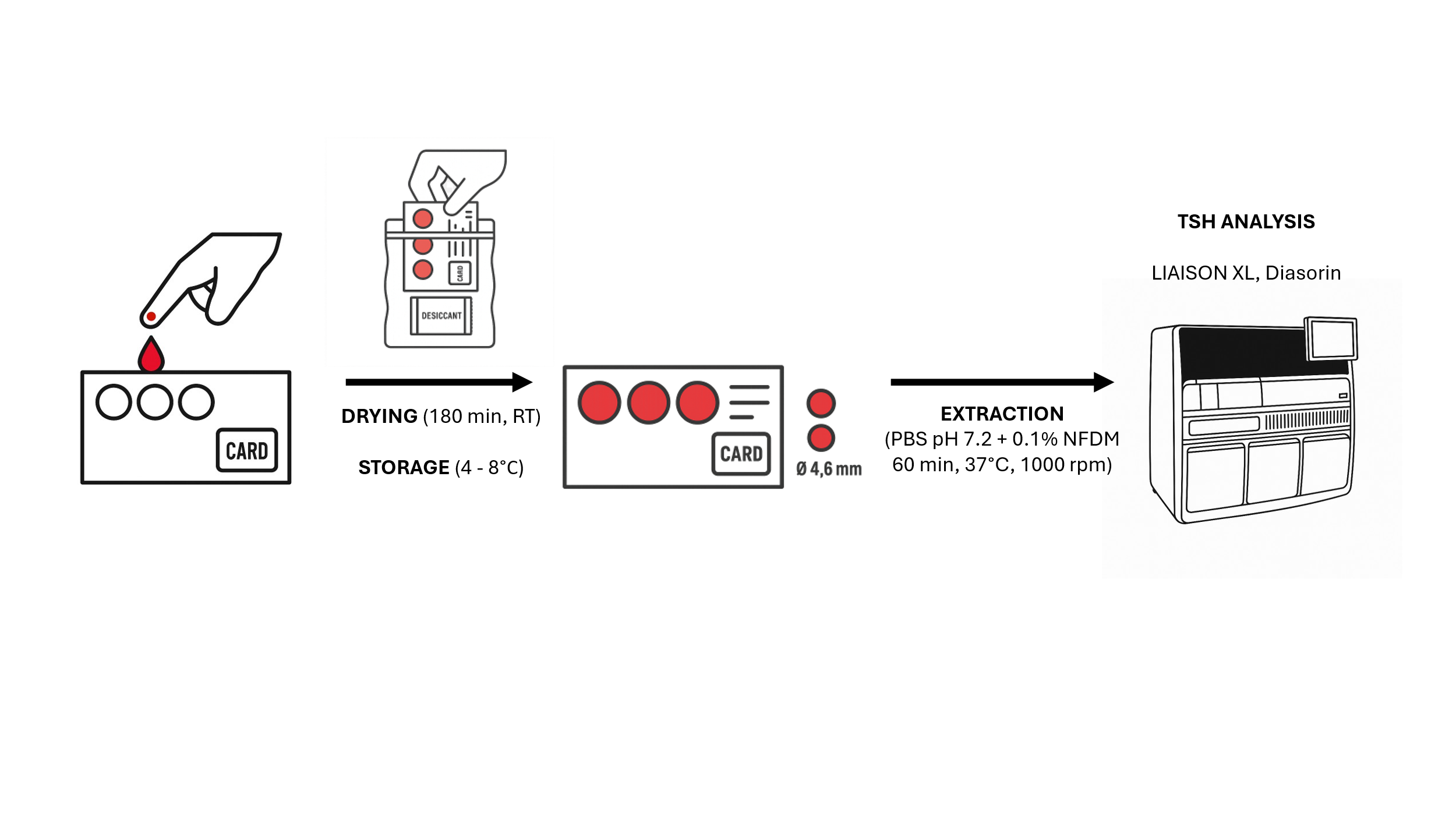


**Figure S3.** Comparison of TSH concentrations between serum (reference method) and CPT B50 microsamples (n = 83). (A) Passing–Bablok regression across the full analytical range. (B) Passing–Bablok regression for TSH <10 mIU/L. (C) Bland–Altman plot showing absolute bias after conversion to serum-equivalent concentrations. (D) Bland–Altman plot showing relative bias (%). The solid line represents the Passing–Bablok regression line; shaded bands indicate 95% confidence intervals. Serum values are plotted on the x-axis. Dashed horizontal lines in panel D indicate predefined acceptance limits (±15%).

Panels A and B present the original microsample concentrations used to derive the regression equations. Serum-equivalent concentrations were subsequently used for Bland–Altman analysis, reference interval transfer, and diagnostic classification. The shaded area represents the 95% confidence interval of the regression line and does not reflect the distribution of individual data points.


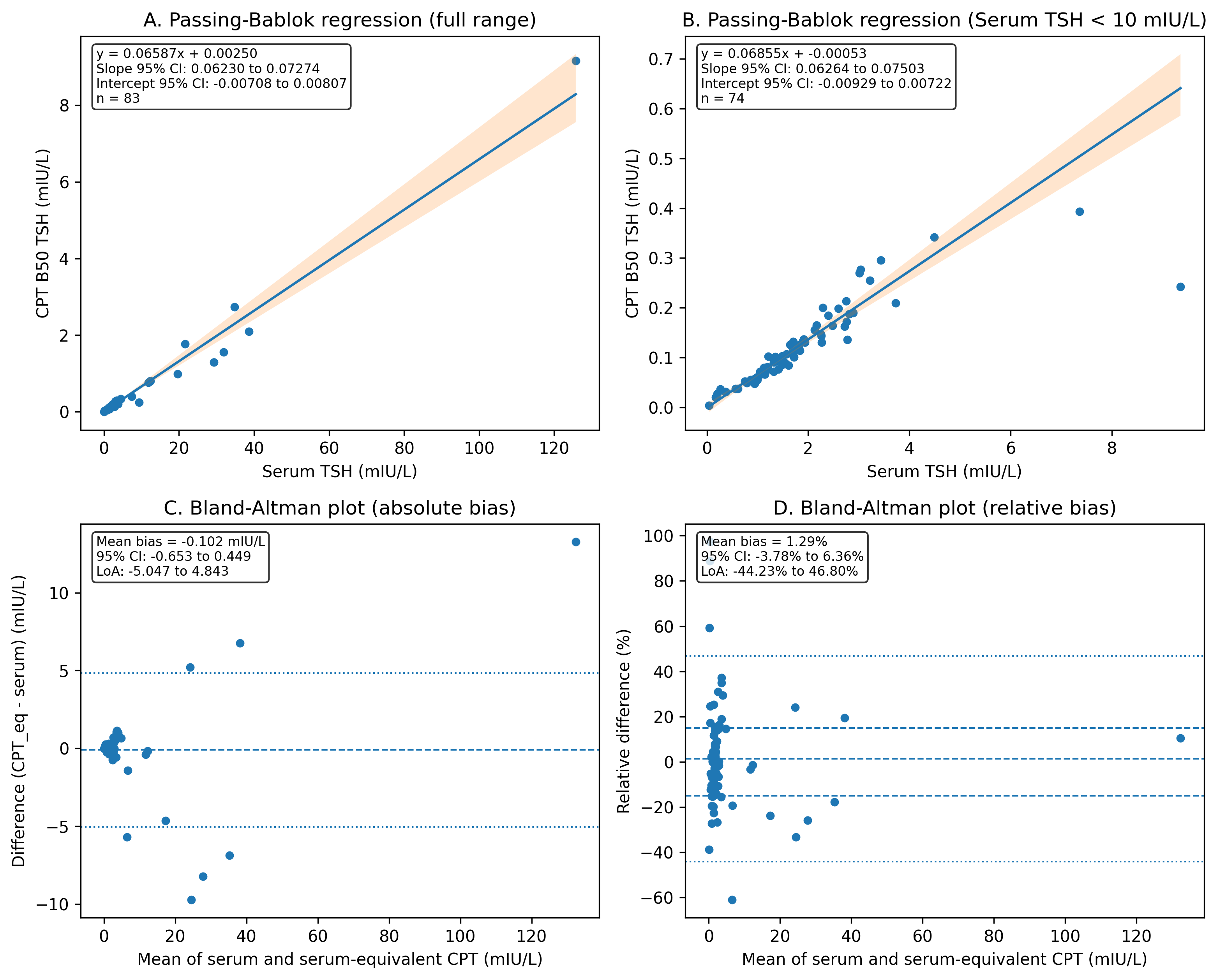


**Figure S4.** Comparison of TSH concentrations between serum (reference method) and DBS microsamples (n = 219). (A) Passing–Bablok regression across the full analytical range. (B) Passing–Bablok regression for TSH <10 mIU/L. (C) Bland–Altman plot showing absolute bias after conversion to serum-equivalent concentrations. (D) Bland–Altman plot showing relative bias (%). The solid line represents the Passing–Bablok regression line; shaded bands indicate 95% confidence intervals. Serum values are plotted on the x-axis. Dashed horizontal lines in panel D indicate predefined acceptance limits (±15%).

Panels A and B present the original microsample concentrations used to derive the regression equations. Serum-equivalent concentrations were subsequently used for Bland–Altman analysis, reference interval transfer, and diagnostic classification. The shaded area represents the 95% confidence interval of the regression line and does not reflect the distribution of individual data points.


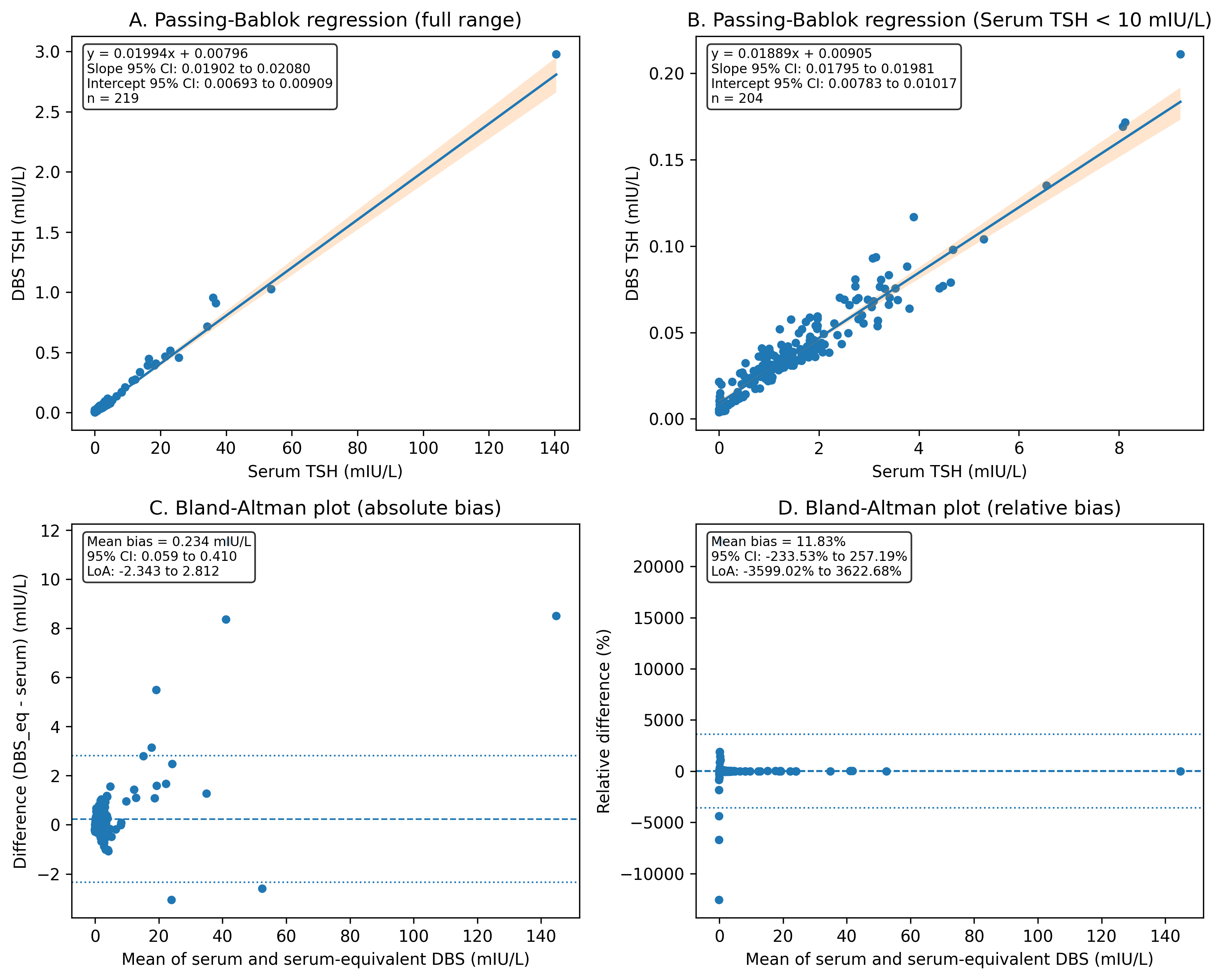

Supplement: Multimedia component 1 [file mmc1.docx]
